# Epidemiological behaviour and interventions of malaria in Niger, 2010–2019: a time-series analysis of national surveillance data

**DOI:** 10.1186/s12936-024-04835-z

**Published:** 2024-01-19

**Authors:** Ali Issakou Malam Tchole, Run-Ze Ye, Qing Xu, Zhen-Wei Li, Jin-Yue Liu, Shan-Shan Wang, Jing Liu, Xiao-Yang Wang, Alassan Maman Bachir, Lin Zhao, Wu-Chun Cao

**Affiliations:** 1 Institute of EcoHealth, School of Public Health, Cheeloo College of Medicine, Shandong University, 44 Wenhua Road, Lixia District, Jinan, 250012 People’s Republic of China; 2 Directorate of Surveillance and Response to Epidemics, Ministry of Public Health, Niamey, Niger; 3 Faculty of Health Sciences, André Salifou University, Zinder, Niger; 4 State Key Laboratory of Pathogen and Biosecurity, Beijing Institute of Microbiology and Epidemiology, Fengtai District, 20 Dong-da Street, Beijing, 100071 People’s Republic of China

**Keywords:** Malaria, Niger, Epidemiological behaviour, Intervention, Incidence

## Abstract

**Background:**

Malaria remains a significant public health concern in Niger, with the number of cases increasing from 592,334 in 2000 to 3,138,696 in 2010. In response, a concerted campaign against the disease has been initiated. However, the implementation of these malaria interventions and their association with epidemiological behaviour remains unclear.

**Methods:**

A time-series study was conducted in Niger from 2010 to 2019. Multiple data sources concerning malaria were integrated, encompassing national surveillance data, Statistic Yearbook, targeted malaria control interventions, and meteorological data. Incidence rate, mortality rate, and case fatality ratio (CFR) by different regions and age groups were analysed. Joinpoint regression models were used to estimate annual changes in malaria. The changes in coverage of malaria interventions were evaluated.

**Results:**

Between 2010 to 2019, the incidence rate of malaria decreased from 249.43 to 187.00 cases per 1,000 population in Niger. Niamey had a high annual mean incidence rate and the lowest CFR, while Agadez was on the contrary. Joinpoint regression analysis revealed a declining trend in malaria incidence for all age groups except the 10–24 years group, and the mortality rate and the CFR initially decreased followed by an increase in all age groups. Niger has implemented a series of malaria interventions, with the major ones being scaled up to larger populations during the study period.

**Conclusions:**

The scale-up of multi-interventions in Niger has significantly reduced malaria incidence, but the rise in mortality rate and CFR addresses the challenges in malaria control and elimination. Malaria endemic countries should enhance surveillance of malaria cases and drug resistance in *Plasmodium*, improve diagnosis and treatment, expand the population coverage of insecticide-treated bed nets and seasonal malaria chemoprevention, and strengthen the management of severe malaria cases.

**Supplementary Information:**

The online version contains supplementary material available at 10.1186/s12936-024-04835-z.

## Background

Malaria is a major public health challenge. With an estimated 247 million new cases and 619,000 deaths in 2021, this disease is a major cause of morbidity and mortality worldwide [1]. In the past two decades, with the aim of accelerating progress towards malaria elimination, interventions for different target groups and settings have been implemented [2]. The incidence rate of malaria thus reduced globally between 2000 and 2019, from 81 to 56.8 cases per 1000 population at risk [3]. Despite tremendous progress in reducing malaria between 2000 and 2015, global progress has stalled in the following years [4]. Many countries, especially those in sub-Saharan Africa, are far from the vision of a world free of malaria set out in the Global Technical Strategy for Malaria 2016–2030 [5]. Among them, four countries including Nigeria, the Democratic Republic of the Congo, Niger, and the United Republic of Tanzania accounted for more than half of global malaria cases [1].

Niger, one of the least developed countries in the world, faces a heavy malaria burden. *Plasmodium falciparum* is the main malaria pathogen in Niger, and *Anopheles gambiae* is the primary vector species (> 96%) [6]. From 2000 to 2010, Niger reported an average of 1,000,805 suspected malaria cases and 1,921 deaths per year [7]. During this period, the number of malaria cases surged five-fold from 592,334 to 3,138,696 [8]. Challenges to malaria elimination in Niger include low socio-economic status, limited health coverage [7], inequality in the distribution of health resources and services [9], frequent climatic shocks, emergence of mosquito insecticide resistance, poor housing, and low education level [10].

To fight against malaria, Niger signed the Abuja Declaration in 2000, committing to reducing malaria mortality by 50% by 2010 [11]. Since 2010, global funding for malaria control has increased in Niger, and a concerted campaign against malaria has been initiated. Niger has introduced seasonal malaria chemoprevention (SMC) for children under 5 years in 2012 and published guidelines for diagnosis and treatment of malaria in 2013. Since 2014, the distribution of bed nets in Niger has expanded from vulnerable populations, such as pregnant women and children under 5 years old, to the whole population [12]. However, the implementation and impact of these interventions require further understanding and evaluation, and their association with epidemiological behaviour over the past decade remains unclear. In this study, multiple sources concerning malaria in Niger, including national surveillance data, Statistic Yearbook, and targeted malaria control interventions, were brought together to establish a comprehensive database. The aim was to investigate the epidemiological behaviour and trends of malaria in Niger from 2010 to 2019, to evaluate the changes in coverage of major interventions and their association with epidemiological behaviour, and thus to develop evidence-based policies in Niger and other sub-Saharan African countries to reduce malaria and save lives.

## Methods

### Study area description

Niger is a landlocked country in West Africa, comprised of seven regions and one capital district: Agadez, Diffa, Dosso, Maradi, Niamey, Tahoua, Tillaberi, and Zinder. As of 2021, the population of Niger was 23,591,981 [13]. Over 80% of Niger’s territory is covered by the Sahara Desert.

Niger is classified as an arid and semi-arid country, with annual rainfall between 100 and 700 mm [14]. It has a long, intense dry season from October to May; and a brief, irregular rainy season from June to September. The density and distribution of vector populations are heavily dependent on the local abundance of rainfall.

### Study design, population and data collection

A time-series study was conducted in Niger from 2010 to 2019. Data on malaria cases, deaths, and population, by age groups (i.e., < 1 year, 1–4 years, 5–9 years, 10–24 years, and ≥ 25 years) and region were extracted from the Statistic Yearbook of Niger (https://www.stat-niger.org/?page_id=500). Annual average population and weekly data on malaria cases and deaths between January 4, 2010 (the first Monday in 2010), and December 29, 2019, were obtained from the Directorate of Surveillance and Response to Epidemics at the Ministry of Public Health (MoH) of Niger.

Meteorological data of Niger, such as temperature and precipitation, was collected from the OpenWeather (https://home.openweathermap.org/). Information on policies, guidelines, initiatives, and interventions related to malaria control and elimination was obtained from the World Health Organization (WHO, https://www.who.int/health-topics/malaria) and the Ministry of Public Health, Population and Social Affairs of Niger (https://www.sante.gouvne.org/projets-et-programmes/). Interventions were divided into the following four categories, vector control, preventive treatment, early diagnosis and prompt treatment, and other supporting measures. Indicators of intervention implementation, including the modelled percentage of population with access to an insecticide-treated bed net (ITN), the average number of children treated with at least one dose of SMC, the number of malaria suspects examined by microscopy and rapid diagnostic test (RDT), and the number of artemisinin-based combination therapy (ACT) treatment courses, were further extracted from the World Malaria Report 2011–2020 published by WHO (https://www.who.int/publications).

### Data analysis

Incidence rate was defined as the number of reported malaria cases divided by the population size during a defined period (cases per 1,000 population); mortality rate as the number of deaths from malaria divided by the population size during a defined period (deaths per 1,000 population); and case fatality ratio (CFR) as the percentage of deaths divided by the number of incident cases (deaths per 1,000 cases). The denominators of annual mean incidence and mortality rate were the sum of the population size for each year during the study period, while the numerators were the total number of malaria cases and deaths during the study period, respectively. Categorical variables were reported as frequency (n) and proportion (%). Seasonal trends of malaria were explored using weekly data from MoH of Niger while the remaining analyses were conducted using data from the Statistic Yearbook. Annual trends of malaria were detected by the Mann–Kendall test.

R software (version 4.0.2, R Foundation for Statistical Computing, Vienna, Austria) was employed for data extraction, cleaning, and analysis. The thematic maps showing geographical distribution of malaria were produced by ArcGIS (version 10.2, ESRI, Redlands, CA, USA). Statistical significance for a two-tailed *P* value was defined as α < 0.05.

### Joinpoint regression analysis

Joinpoint regression is known to be an effective tool for inferring changes in trends over time [15], while interrupted time series analysis has been identified as a common approach to explore the effectiveness of the single intervention within a short period [16]. This study aimed to investigate the changes in malaria incidence and mortality over time, and to analyse the relationship between malaria changes and the implementation of various malaria interventions concurrently or consecutively over a 10-year period. Therefore, the annual percentage change (APC) of malaria from 2010 to 2019 was estimated, stratified by age groups using joinpoint regression. The long-term trend of malaria and the potential effects of multiple malaria interventions were then inferred.

Additionally, the incidence was used as the outcome rather than total case count in the joinpoint regression model because of substantial changes in the total population of Niger from 2010 to 2019. The permutation test was used to identify the number of significant joinpoints, and each test was assessed using the Monte Carlo method. A grid search method (GSM) was employed to fit the segmented regression function and a maximum number of two segments (one joinpoint) was applied in the models. By using the year as a regression variable, the joinpoint regression analysis estimated the APC in rates between change points, with its 95% confidence intervals (CI). The Z-test was used to assess whether an APC was significantly different from zero. If the APC was significant (P < 0.05), the incidence trend was identified as an increase or decrease; otherwise, the incidence was maintained stable. The joinpoint regression analyses were conducted using Joinpoint Regression Program (version 4.8.0.1, National Cancer Institute, MD, USA).

## Results

### Epidemiological behaviour of malaria in Niger

A total of 37,052,982 malaria cases were reported from 2010 to 2019, with an annual mean incidence rate of 199.44 cases per 1,000 population. Among all cases, 33,111 deaths were recorded, resulting in an annual mean mortality rate of 0.18 deaths per 1,000 population and a CFR of 0.89 deaths per 1,000 cases. During the study period, the incidence rate decreased significantly by 25.03%, from 249.43 cases per 1,000 population in 2010 to 187.00 cases per 1,000 population in 2019 (Mann-Kendall test, *P* = 0.02). The mortality rate showed a declining trend with mild fluctuations. From 2010 to 2016, the CFR decreased by 74.12% from 1.70 to 0.44 deaths per 1000 cases. However, the decreasing trend changed after 2016 (Table 1).

**Table 1:** Annual incidence rate, mortality rate, and case fatality ratio in Niger from 2010 to 2019

Year	Incidence rate, per 1,000 population (95% CI)	Mortality rate, per 1,000 population (95% CI)	Case fatality ratio, per 1,000 cases (95% CI)
2010	249.43 (249.22, 249.64)	0.42 (0.41, 0.43)	1.70 (1.66, 1.74)
2011	190.34 (190.15, 190.53)	0.17 (0.16, 0.17)	0.88 (0.85, 0.91)
2012	267.00 (266.79, 267.21)	0.18 (0.18, 0.19)	0.69 (0.67, 0.71)
2013	206.86 (206.67, 207.05)	0.13 (0.12, 0.13)	0.61 (0.59, 0.64)
2014	202.56 (202.37, 202.74)	0.19 (0.18, 0.20)	0.94 (0.91, 0.97)
2015	201.30 (201.12, 201.48)	0.09 (0.09, 0.09)	0.44 (0.42, 0.47)
2016	185.54 (185.37, 185.72)	0.08 (0.08, 0.09)	0.44 (0.42, 0.46)
2017	159.42 (159.26, 159.57)	0.13 (0.12, 0.13)	0.81 (0.78, 0.84)
2018	170.43 (170.27, 170.59)	0.17 (0.17, 0.18)	1.03 (0.99, 1.06)
2019	187.00 (186.84, 187.17)	0.25 (0.24, 0.25)	1.31 (1.28, 1.35)
Overall	199.44 (199.38, 199.49)	0.18 (0.18, 0.18)	0.89 (0.88, 0.90)

*CI*, Confidence interval

**Fig. 1:**
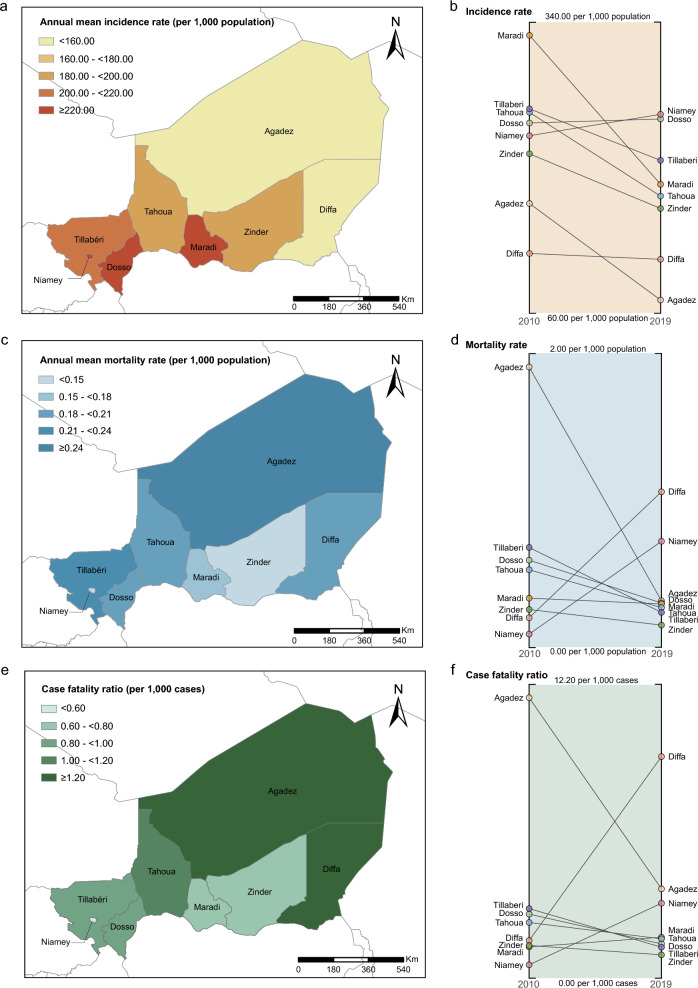
Spatial (**a**, **c**, and **e**) and temporal (**b**, **d**, and **f**) distribution of malaria in Niger

Dosso had the highest annual mean incidence rate (236.86 cases per 1,000 population) across Niger, followed by Niamey (227.51 cases per 1,000 population) and Maradi (227.30 cases per 1000 population) (Fig. 1a). Although Agadez had the lowest annual mean incidence rate (105.46 cases per 1000 population), it had the highest annual mean mortality rate (0.26 deaths per 1,000 population) and CFR (2.44 deaths per 1,000 cases) (Fig. 1c, e). Additionally, the regions with the lowest annual mean mortality rate and CFR were Zinder (0.11 deaths per 1,000 population) and Niamey (0.57 deaths per 1,000 cases), respectively. In 2010, Maradi had the highest annual incidence rate of 323.38 cases per 1,000 population (95% CI: 322.87, 323.90), which was 2.7 times higher than Diffa (119.94 cases per 1,000 population, 95% CI: 119.03, 120.85) (Fig. 1b, Additional file 1: Table S1). During the 10-year period, the annual mortality rate and the CFR decreased in all regions except Diffa and Niamey (Fig. 1d, f, Additional file 1: Fig. S1, S2, S3), and the decline was particularly significant in Agadez.

**Fig. 2:**
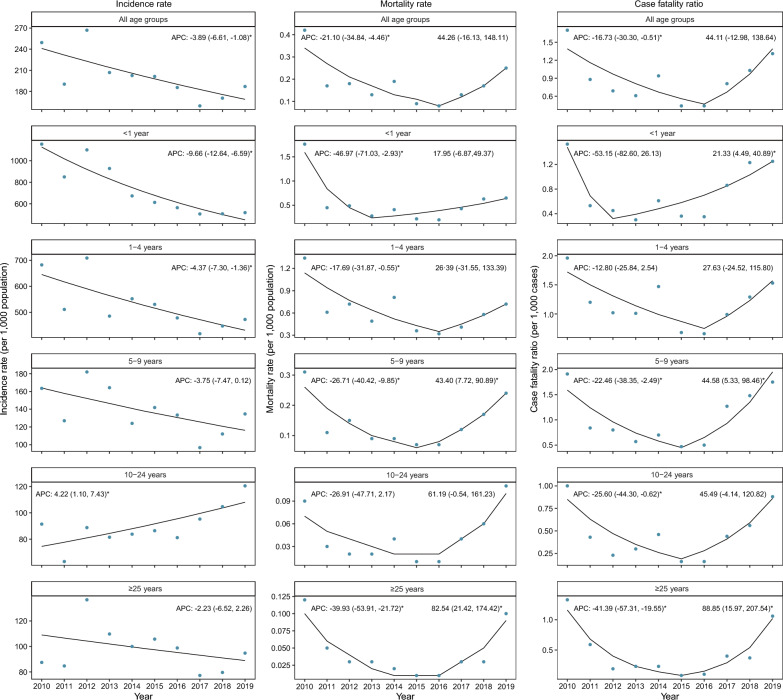
Trends of the incidence rate, mortality rate, and case fatality ratio by age groups in Niger. Joinpoint regression models were used to estimate the annual percentage change (APC) of malaria from 2010 to 2019 stratified by age groups. Blue points indicate the observed rate of malaria and the curve indicates fitted patterns by blue points. *APC is significantly different from zero at two-sided *P* < 0.05

Among all age groups, 59.4% of malaria cases were children under 5 years. Infants aged < 1 year showed the highest incidence rate at 706.40 cases per 1,000 population, followed by 10–24 years age group (524.53 cases per 1,000 population). Children in the age group of 1–4 years had the highest mortality rate (0.63 deaths per 1,000 population) and CFR (1.19 deaths per 1,000 cases), while adults aged ≥ 25 years had the lowest (0.04 deaths per 1,000 population and 0.43 deaths per 1,000 cases, respectively) (Additional file 1: Table S2). Joinpoint regression analysis indicated an annual percentage change of -3.89% (95% CI -6.61, -1.08, *P* = 0.01) in incidence rate (Fig. 2). Significant decreasing trends were identified in both < 1 year (APC = -9.66%, *P* < 0.001) and 1–4 years (APC = -4.37%, *P* = 0.01) age groups. Surprisingly, the incidence of malaria in the 10–24 years age group showed a significant increasing trend (APC = 4.22%, *P* = 0.01). The trends of mortality and CFR were significantly and linearly decreasing from 2010 to 2016 (*P* < 0.05), and increased in the period 2017–2019 (*P* > 0.05). Similar changing pattern was observed in different age categories, however, the increasing turn points varied across different age groups.

**Fig. 3:**
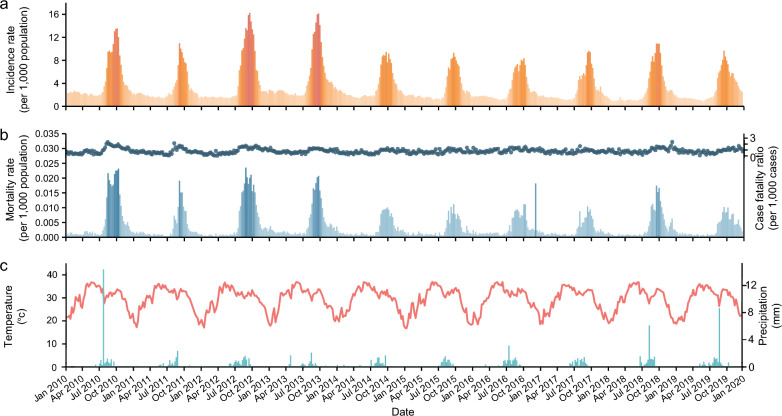
Seasonal trends of malaria and associated meteorological factors in Niger. **a** Monthly incidence rate; **b** Monthly mortality rate and case fatality ratio, indicated by blue shades and points, respectively; **c** Monthly temperature and precipitation, indicated by the line of orange red and the shades of cyan-blue, respectively

Figure 3a, plotted by weekly malaria data, depicts that the temporal pattern of malaria epidemic in Niger was seasonal and cyclical. Though malaria occurred in all seasons, the highest incidence rate was observed from July to October every year. The mortality rate showed similar fluctuations to incidence rate, while the CFR exhibited a relatively stable trend (Fig. 3b). As displayed in Fig. 3c, Niger’s temperature remained consistently high across all seasons. The rainfall demonstrated interannual fluctuations and was predominantly concentrated in the summer season, spanning from June to September, which coincided with the peak of malaria incidence.

### Implementation and change of malaria interventions in Niger

**Fig. 4:**
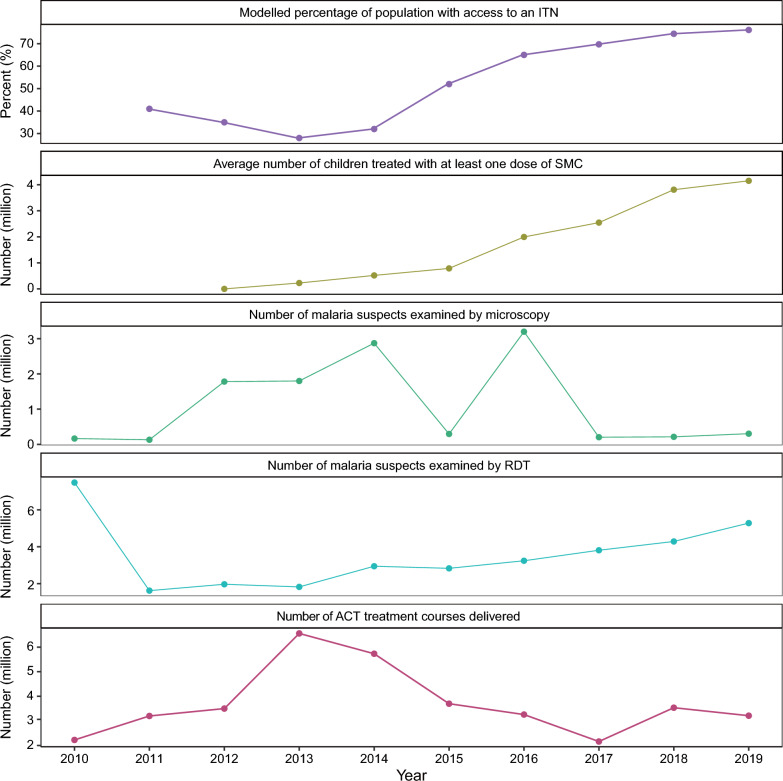
Time trends of indicators related to interventions for malaria control and elimination in Niger. ITN, insecticide-treated bed net; SMC, seasonal malaria chemoprevention; RDT, rapid diagnostic test; ACT, artemisinin-based combination therapy

To further explore the efficacy of major malaria interventions in Niger, policies, guidelines, initiatives, and measures of malaria published or implemented by WHO and Niger since 2010 were collected (Additional file 1: Figure S4). From 2010 to 2015, the focus of malaria interventions was mainly on preventive treatment and early diagnosis and prompt treatment. Afterward, the focus shifted to vector control and other supporting measures. Niger issued the National Malaria Control Programme (NMCP) in 2011 and 2016 to promote malaria control and achieve the vision of "A Niger Without Malaria". From 2013 to 2019, the modelled percentage of population with access to an ITN doubled from 28.00% to 76.10%, and the average number of children treated with at least one dose of SMC increased from 225,970 to 4,151,103 (Fig. 4).

Correspondingly, the incidence rate of malaria exhibited a significant declining trend as modelled by joinpoint regression (Fig. 2). In 2010, the number of malaria suspects examined by RDT reached a peak at 747,667 persons. Subsequently, there was a sharp decline in this number, followed by a gradual upward trend from 2013, reaching 5,279,843 individuals by 2019.

## Discussion

This is the first comprehensive study exploring the epidemiological characteristics, long-term trends, and changes in intervention coverage of malaria in Niger using multi-source data. Coinciding with the implementation and expansion of the government’s multi-pronged malaria interventions, there was a decrease in malaria incidence rate from 249.43 to 187.00 cases per 1000 population between 2010 and 2019. The magnitude and patterns of decline varied by region and age group. Additionally, the mortality and the CFR showed a decreasing trend from 2010 to 2016, but this trend changed after 2016.

Niger's diverse climate and rainfall patterns contribute to the endemic nature of malaria, with the country divided into four malaria strata [17]. The spatial distribution of malaria incidence obtained from the study was almost consistent with that reported in the NMSP 2017–2023 [17]. In the findings, the highest annual mean incidence rate of malaria was reported in Dosso (236.86 cases per 1,000 population), followed by Niamey (227.51 cases per 1,000 population) and Maradi (227.30 cases per 1,000 population). These regions experience high annual precipitations, and correspondingly high incidence rates of malaria, showing strong seasonality. Although Niamey, the capital of Niger, had a very high annual mean incidence rate, its CFR was the lowest (0.57 deaths per 1,000 cases). Niamey is a rapidly growing city with high accessibility and quality of medical care [18]. According to the NMCP 2017–2021 [19], the coverage of health services in Niamey is up to 98.39%, partially explaining its low mortality rate and CFR of malaria.

Agadez, where the majority of districts are situated in a region with very low or sporadic malaria transmission according to NMSP 2017–2023 [17], exhibited the highest annual mean mortality rate (0.26 deaths per 1,000 population) and CFR (2.44 deaths per 1,000 cases). The possible reasons are as follows. First, due to the poor infrastructure and lack of paved roads, access to health facilities is difficult in eastern Agadez, especially during the rainy season [20]. Second, terrorist attacks and other political-economic factors have caused a significant influx of migrant populations in Agadez [21], resulting in poor access to healthcare. Third, healthcare providers in Agadez have limited capacity to manage malaria cases [22]. Therefore, the harsh climate, inadequate infrastructure, large migrant populations, and low management capacity make it more difficult for residents and cases to receive health services in Agadez, consequently contributing to the higher mortality rate and CFR from malaria. Moreover, major malaria interventions, such as ITN mass distribution campaigns and SMC campaigns were absent in Agadez, making malaria control more challenging in this region. Hence, establishing more health facilities, improving the accessibility and effectiveness of health-care services, and enhancing the management capacity of healthcare providers are beneficial to reduce malaria mortality [23].

Joinpoint regression analysis revealed a declining trend in incidence rate of malaria, which might be attributed to the following interventions: introduction of SMC, improvements of diagnostic accuracy and treatment, and large-scale distribution of mosquito nets. The results showed a significant decrease in malaria incidence among children under 5 years, who accounted for 59.4% of malaria cases during the study period. In 2012, the WHO recommended SMC as an effective tool for children aged 3 to 59 months to fight against malaria. With the support of Doctors Without Borders, the NMCP introduced SMC into Niger for malaria prevention and treatment in 2013 [24], and the coverage rates of SMC varied from 59 to 99% in seven administrative regions of Niger by 2016 [25]. Multiple studies have pointed out that SMC is a low-cost and highly effective intervention to prevent malaria during high transmission seasons [26], and could avert tens of thousands of childhood deaths annually [27]. Expanding population coverage of SMC in Niger with sustained funding could further enhance its cost-effectiveness.

In 2013, the WHO provided a comprehensive roadmap for scaling up diagnostic testing through the issue of *Universal access to malaria diagnostic testing: An operational manual*, and Niger published guidelines for malaria diagnosis and treatment, outlining a series of interventions to improve diagnostic accuracy and treatment [28]. In Africa, presumptive diagnosis was the predominant diagnostic approach, leading to an overdiagnosis of malaria cases. However, improvements in malaria testing capabilities have made it possible to identify malaria cases more accurately than before [29]. Therefore, it is necessary to strengthen the implementation of related guidelines and confirm all suspected malaria cases through biological diagnosis.

In 2014, the WHO issued recommendations for achieving universal coverage with LLINs. Since then, the distribution of bed nets in Niger has expanded from vulnerable populations, such as pregnant women and children under 5 years old, to the entire population [12]. By 2019, the modelled percentage of population with access to an ITN in Niger had reached 76.10%. ITNs are considered to be the most significant contributor to vector control by far [30]. Monitoring the durability and bio-efficacy of ITNs is an important task at this stage, as it helps in planning the replacement of worn-out nets, and understanding the influencing factors of bed net durability [31].

Surprisingly, the incidence rate of malaria in the 10–24 years age group increased, when other age groups were all on a downward trend. One of the possible explanations would be that adolescents and young adults of this age are economically and socially active and are less likely to sleep under mosquito nets compared with children. Another possible reason is that the younger children are the target age group for SMC while individuals aged 10–24 years are not the priority for protection. Targeted interventions for this age group are thus needed, including improving their knowledge on the etiology and prevention of malaria, and increasing their accessibility to SMC and ITNs.

A substantial reduction in mortality rate and CFR over the previous years of the study was possibly due to the scale-up of ACT, which is a recommended first-line treatment for malaria in endemic countries. Niger expanded the supply of ACT in 2008. The pilot phase of the Affordable Medicines Facility-malaria was launched in eight countries in 2010, increasing the availability of affordable ACT in Niger [32].

Moreover, in 2013, the WHO launched the Rapid Access Expansion (RAcE) programme in five countries, including Niger, to increase the coverage of Integrated Community Case Management (iCCM) interventions among children aged 2–59 months [31]. Initially implemented in four health districts in 2013 in Niger, the coverage of iCCM expanded over time, reaching 35 districts by 2019 [34]. Research indicated that the implementation of iCCM was associated with an average 10% reduction in mortality rates among children under five years, and it could notably decrease child mortality in regions with limited access to health facility services [33].

However, the World Malaria Report 2022 showed that the global mortality rate of malaria has remained unchanged since 2016 [1]. In Niger, both the mortality rate and the CFR have increased since 2016, warranting attention. The possible reasons may be due to the neglect of severe malaria and lower public health awareness. Severe malaria is a major cause of preventable childhood death in tropical countries [35], but it is increasingly overlooked by donors and policy makers. Its pathobiology and clinical management are rarely discussed in international conferences, potentially contributing to a higher CFR [35, 36]. From 2016 to 2019, there was only one policy for malaria diagnosis and treatment, *Guide to G6PD deficiency rapid diagnostic testing to support P. vivax radical cure*. To reduce mortality, it is essential to enhance case management, including early administration of artesunate and broad-spectrum antibiotics to all individuals with suspected severe malaria. The increasing CFR may be associated with lower public health awareness, suboptimal healthcare-seeking behaviour, and delays in diagnosis and/or treatment [37]. Ensuring consistent and timely access to antimalarial treatments, especially in remote areas, remains a persistent issue. Furthermore, socio-economic factors, including poverty and limited access to healthcare, continue to hinder effective malaria control. To address these challenges and boost malaria elimination, a comprehensive approach and adaptation strategies to the evolving landscape of the disease are imperative, integrating health system strengthening, community engagement, and continued research to inform evidence-based interventions.

Additionally, loss of efficacy of ACT could have terrible consequences, as chloroquine resistance occurred at the end of the twentieth century, which led to a devastating increase in malaria deaths [38]. Current surveys in Niger suggest that the first line ACT is still viable [39]. However, resistance to ACT has emerged in Africa due to the selective pressure on parasites from poor adherence and excessive use of injectable artesunate [40]. The primary task at present is to enhance surveillance on *Plasmodium* drug resistance, and to develop new approaches to maintain long-term efficacy of anti-malarial regimens.

There are several limitations in this study. First, due to limited medical resources, surveillance and reporting of malaria in Niger might be incomplete, resulting in an underestimated disease burden. Second, the lack of detailed case information makes it difficult to distinguish new malaria cases from recurrent ones, potentially leading to inaccuracies in incidence estimation and risk assessment. Future studies with more precise and targeted data will be needed to confirm the findings. Third, the lack of monthly malaria data stratified by age groups hindered the ability to identify general trends of malaria over time using joinpoint regression. Moreover, although dozens of global and national, small- or large-scale interventions were implemented in Niger, only changes in the coverage of several important public health and clinical interventions and their associations with epidemiological behaviour were evaluated.

## Conclusions

In conclusion, the scale-up of multiple malaria interventions, involving improvements in diagnosis and treatment, preventive treatment, and vector control measures, has resulted in significant reductions in malaria incidence from 2010 to 2019. However, the rebound mortality rate and CFR highlight the challenges in achieving "A Niger Without Malaria" and underscores the need to adapt strategies to address these evolving challenges and make sustainable progress in malaria control.

The study provides evidence-based guidance for Niger and other sub-Saharan African countries in the fight against malaria. Authorities and healthcare workers in these countries should enhance surveillance of malaria cases and drug resistance in *Plasmodium*, expand the coverage of ITNs and SMC, improve diagnosis and treatment, and strengthen the management of severe malaria cases.

### Supplementary Information

**Additional file 1: Table S1.** Annual incidence rate, mortality rate, and case fatality ratio by region in Niger from 2010 to 2019. **Table S2.** Annual epidemiological indicators of malaria by age group in Niger from 2010 to 2019. **Figure S1.** Map of malaria incidence rate in Niger from 2010 to 2019. **Figure S2.** Map of malaria mortality rate in Niger from 2010 to 2019. **Figure S3.** Map of malaria case fatality ratio rate in Niger from 2010 to 2019. **Figure S4.** Interventions issued by the World Health Organization (WHO) and Niger.

## Data Availability

The datasets used and/or analysed during the current study are available from the corresponding author on reasonable request.

## Abbreviations

ACT: Artemisinin-based combination therapy
APC: Annual percentage change
CFR: Case fatality ratio
CI: Confidence interval
ICCM: Integrated Community Case Management
ITNs: Insecticide-treated bed nets
LLINs: Long-lasting insecticidal nets
MoH: Ministry of Public Health
NMCP: National Malaria Control Programme
RDT: Rapid diagnostic test
SMC: Seasonal malaria chemoprevention
WHO: World Health Organization
