# Supplementary material for: Epidemiological behaviour and interventions of malaria in Niger, 2010–2019: a time-series analysis of national surveillance data
Source: Malar J. 2024 Jan 19;23:30. doi: 10.1186/s12936-024-04835-z (PMC10799420; doi:10.1186/s12936-024-04835-z)
Supplement: Supplementary file 1 — Additional file 1: Table S1. Annual incidence rate, mortality rate, and case fatality ratio by region in Niger from 2010 to 2019. Table S2. Annual epidemiological indicators of malaria by age group in Niger from 2010 to 2019. Figure S1. Map of malaria incidence rate in Niger from 2010 to 2019. Figure S2. Map of malaria mortality rate in Niger from 2010 to 2019. Figure S3. Map of malaria case fatality ratio rate in Niger from 2010 to 2019. Figure S4. Interventions issued by the World Health Organization (WHO) and Niger. [file 12936_2024_4835_MOESM1_ESM.docx]

**Additional file to**: Epidemiological behavior and interventions of malaria in Niger, 2010-2019: a time-series analysis of national surveillance data

| **Page** | **Item** |
| --- | --- |
| 1 | **Table S1. Annual incidence rate, mortality rate, and case fatality ratio by region in Niger from 2010 to 2019.** |
| 4 | **Fig. S1. Map of malaria incidence rate in Niger from 2010 to 2019.** |
| 5 | **Fig. S2. Map of malaria mortality rate in Niger from 2010 to 2019.** |
| 6 | **Fig. S3. Map of malaria case fatality ratio rate in Niger from 2010 to 2019.** |
| 7 | **Table S2. Annual epidemiological indicators of malaria by age group in Niger from 2010 to 2019.** |
| 10 | **Fig. S4. Interventions issued by the World Health Organization (WHO) and Niger.** |

**Table S1. Annual incidence rate, mortality rate, and case fatality ratio by region in Niger from 2010 to 2019.**

| **Region** | **Year** | **Incidence rate, per 1,000 population (95% CI)** | **Mortality rate, per 1,000 population (95% CI)** | **Case fatality ratio, per 1,000 cases (95% CI)** |
| --- | --- | --- | --- | --- |
| Agadez |  |  |  |  |
|  | 2010 | 166.54 (165.51, 167.58) | 1.95 (1.83, 2.07) | 11.71 (10.98, 12.44) |
|  | 2011 | 97.57 (96.77, 98.37) | 0.14 (0.10, 0.17) | 1.39 (1.07, 1.71) |
|  | 2012 | 133.10 (132.20, 134.01) | 0.07 (0.05, 0.09) | 0.51 (0.35, 0.68) |
|  | 2013 | 69.00 (68.34, 69.67) | 0.12 (0.09, 0.15) | 1.71 (1.30, 2.12) |
|  | 2014 | 109.87 (109.02, 110.72) | 0.01 (0.00, 0.02) | 0.09 (0.01, 0.16) |
|  | 2015 | 108.49 (107.66, 109.32) | 0.01 (0.00, 0.03) | 0.14 (0.04, 0.23) |
|  | 2016 | 94.31 (93.54, 95.07) | 0.06 (0.04, 0.08) | 0.59 (0.38, 0.80) |
|  | 2017 | 73.92 (73.25, 74.59) | 0.04 (0.03, 0.06) | 0.58 (0.35, 0.81) |
|  | 2018 | 134.11 (133.25, 134.97) | 0.08 (0.06, 0.11) | 0.62 (0.45, 0.79) |
|  | 2019 | 76.54 (75.87, 77.21) | 0.26 (0.22, 0.30) | 3.39 (2.86, 3.92) |
| Diffa |  |  |  |  |
|  | 2010 | 119.94 (119.03, 120.85) | 0.14 (0.10, 0.17) | 1.14 (0.87, 1.41) |
|  | 2011 | 72.75 (72.04, 73.47) | 0.08 (0.06, 0.11) | 1.14 (0.79, 1.48) |
|  | 2012 | 179.19 (178.16, 180.23) | 0.13 (0.10, 0.16) | 0.70 (0.53, 0.87) |
|  | 2013 | 130.07 (129.18, 130.97) | 0.10 (0.07, 0.12) | 0.74 (0.54, 0.94) |
|  | 2014 | 147.94 (147.06, 148.82) | 0.04 (0.02, 0.05) | 0.26 (0.16, 0.36) |
|  | 2015 | 94.49 (93.78, 95.20) | 0.07 (0.05, 0.10) | 0.79 (0.57, 1.02) |
|  | 2016 | 142.41 (141.58, 143.23) | 0.05 (0.03, 0.07) | 0.36 (0.24, 0.48) |
|  | 2017 | 108.17 (107.44, 108.90) | 0.02 (0.01, 0.03) | 0.21 (0.11, 0.32) |
|  | 2018 | 129.72 (128.94, 130.50) | 0.07 (0.05, 0.09) | 0.53 (0.38, 0.68) |
|  | 2019 | 114.53 (113.81, 115.26) | 1.05 (0.97, 1.12) | 9.14 (8.50, 9.79) |
| Dosso |  |  |  |  |
|  | 2010 | 241.66 (241.08, 242.24) | 0.55 (0.52, 0.58) | 2.29 (2.16, 2.42) |
|  | 2011 | 241.31 (240.73, 241.88) | 0.19 (0.17, 0.21) | 0.78 (0.71, 0.86) |
|  | 2012 | 301.62 (301.02, 302.23) | 0.18 (0.17, 0.20) | 0.61 (0.55, 0.67) |
|  | 2013 | 263.17 (262.60, 263.74) | 0.12 (0.11, 0.14) | 0.46 (0.41, 0.52) |
|  | 2014 | 281.84 (281.23, 282.45) | 0.13 (0.12, 0.15) | 0.47 (0.41, 0.52) |
|  | 2015 | 269.95 (269.36, 270.55) | 0.15 (0.13, 0.17) | 0.55 (0.49, 0.61) |
|  | 2016 | 255.80 (255.22, 256.37) | 0.11 (0.10, 0.12) | 0.43 (0.37, 0.48) |
|  | 2017 | 101.20 (100.83, 101.57) | 0.20 (0.18, 0.21) | 1.95 (1.78, 2.12) |
|  | 2018 | 195.64 (195.16, 196.12) | 0.19 (0.17, 0.20) | 0.95 (0.86, 1.03) |
|  | 2019 | 245.35 (244.83, 245.87) | 0.24 (0.22, 0.26) | 0.99 (0.92, 1.07) |
| Maradi |  |  |  |  |
|  | 2010 | 323.38 (322.87, 323.90) | 0.28 (0.26, 0.30) | 0.86 (0.80, 0.92) |
|  | 2011 | 209.20 (208.76, 209.65) | 0.15 (0.14, 0.16) | 0.72 (0.66, 0.79) |
|  | 2012 | 269.09 (268.62, 269.57) | 0.16 (0.14, 0.17) | 0.59 (0.54, 0.64) |
|  | 2013 | 159.18 (158.80, 159.56) | 0.14 (0.12, 0.15) | 0.86 (0.78, 0.93) |
|  | 2014 | 259.35 (258.89, 259.80) | 0.30 (0.28, 0.32) | 1.17 (1.10, 1.24) |
|  | 2015 | 252.85 (252.41, 253.29) | 0.06 (0.05, 0.07) | 0.23 (0.20, 0.26) |
|  | 2016 | 219.04 (218.62, 219.45) | 0.06 (0.06, 0.07) | 0.29 (0.25, 0.32) |
|  | 2017 | 212.15 (211.75, 212.54) | 0.12 (0.11, 0.13) | 0.57 (0.52, 0.62) |
|  | 2018 | 209.99 (209.60, 210.37) | 0.26 (0.24, 0.27) | 1.22 (1.15, 1.29) |
|  | 2019 | 184.45 (184.09, 184.82) | 0.24 (0.22, 0.25) | 1.28 (1.20, 1.36) |
| Niamey |  |  |  |  |
|  | 2010 | 229.79 (229.05, 230.52) | 0.02 (0.01, 0.03) | 0.08 (0.05, 0.11) |
|  | 2011 | 192.50 (191.83, 193.17) | 0.06 (0.04, 0.07) | 0.30 (0.23, 0.36) |
|  | 2012 | 218.70 (218.00, 219.39) | 0.02 (0.02, 0.03) | 0.11 (0.08, 0.15) |
|  | 2013 | 234.90 (234.21, 235.60) | 0.01 (0.00, 0.01) | 0.04 (0.02, 0.06) |
|  | 2014 | 221.39 (220.60, 222.18) | 0.01 (0.00, 0.01) | 0.03 (0.01, 0.05) |
|  | 2015 | 260.60 (259.77, 261.42) | 0.01 (0.01, 0.02) | 0.05 (0.03, 0.08) |
|  | 2016 | 242.07 (241.28, 242.86) | 0.01 (0.01, 0.02) | 0.05 (0.02, 0.08) |
|  | 2017 | 213.67 (212.94, 214.40) | 0.09 (0.07, 0.10) | 0.40 (0.33, 0.48) |
|  | 2018 | 217.20 (216.48, 217.93) | 0.36 (0.32, 0.39) | 1.64 (1.49, 1.79) |
|  | 2019 | 249.71 (248.96, 250.46) | 0.69 (0.64, 0.73) | 2.76 (2.58, 2.94) |
| Tahoua |  |  |  |  |
|  | 2010 | 251.58 (251.06, 252.09) | 0.48 (0.46, 0.51) | 1.92 (1.82, 2.03) |
|  | 2011 | 193.19 (192.72, 193.65) | 0.16 (0.14, 0.17) | 0.82 (0.75, 0.90) |
|  | 2012 | 295.16 (294.63, 295.68) | 0.27 (0.25, 0.29) | 0.91 (0.85, 0.98) |
|  | 2013 | 241.60 (241.12, 242.09) | 0.13 (0.12, 0.15) | 0.56 (0.50, 0.61) |
|  | 2014 | 181.23 (180.82, 181.65) | 0.41 (0.39, 0.43) | 2.28 (2.16, 2.40) |
|  | 2015 | 167.05 (166.66, 167.44) | 0.06 (0.05, 0.07) | 0.38 (0.33, 0.43) |
|  | 2016 | 187.05 (186.65, 187.45) | 0.09 (0.08, 0.10) | 0.49 (0.44, 0.54) |
|  | 2017 | 152.50 (152.15, 152.85) | 0.13 (0.12, 0.14) | 0.83 (0.75, 0.90) |
|  | 2018 | 133.76 (133.43, 134.09) | 0.16 (0.15, 0.17) | 1.18 (1.09, 1.27) |
|  | 2019 | 173.44 (173.08, 173.79) | 0.21 (0.20, 0.22) | 1.21 (1.13, 1.29) |
| Tillaberi |  |  |  |  |
|  | 2010 | 254.87 (254.34, 255.40) | 0.64 (0.61, 0.68) | 2.53 (2.41, 2.65) |
|  | 2011 | 216.00 (215.51, 216.50) | 0.17 (0.15, 0.18) | 0.78 (0.71, 0.86) |
|  | 2012 | 266.23 (265.71, 266.75) | 0.24 (0.22, 0.26) | 0.89 (0.82, 0.96) |
|  | 2013 | 252.18 (251.68, 252.69) | 0.21 (0.20, 0.23) | 0.84 (0.77, 0.91) |
|  | 2014 | 203.36 (202.89, 203.83) | 0.13 (0.12, 0.14) | 0.64 (0.57, 0.70) |
|  | 2015 | 252.43 (251.93, 252.93) | 0.21 (0.20, 0.23) | 0.84 (0.78, 0.91) |
|  | 2016 | 193.02 (192.57, 193.47) | 0.15 (0.14, 0.16) | 0.78 (0.70, 0.85) |
|  | 2017 | 169.54 (169.13, 169.95) | 0.12 (0.11, 0.13) | 0.72 (0.65, 0.79) |
|  | 2018 | 160.91 (160.52, 161.30) | 0.17 (0.15, 0.18) | 1.04 (0.96, 1.13) |
|  | 2019 | 206.81 (206.38, 207.23) | 0.18 (0.16, 0.19) | 0.85 (0.78, 0.92) |
| Zinder |  |  |  |  |
|  | 2010 | 212.91 (212.44, 213.37) | 0.20 (0.18, 0.21) | 0.92 (0.85, 1.00) |
|  | 2011 | 143.52 (143.12, 143.91) | 0.24 (0.23, 0.26) | 1.69 (1.57, 1.81) |
|  | 2012 | 273.63 (273.14, 274.12) | 0.19 (0.17, 0.20) | 0.69 (0.63, 0.74) |
|  | 2013 | 170.07 (169.66, 170.48) | 0.10 (0.09, 0.11) | 0.57 (0.51, 0.64) |
|  | 2014 | 139.13 (138.77, 139.48) | 0.07 (0.06, 0.08) | 0.50 (0.44, 0.56) |
|  | 2015 | 120.44 (120.11, 120.76) | 0.05 (0.05, 0.06) | 0.43 (0.38, 0.49) |
|  | 2016 | 113.52 (113.21, 113.82) | 0.05 (0.05, 0.06) | 0.46 (0.40, 0.53) |
|  | 2017 | 146.19 (145.86, 146.53) | 0.14 (0.13, 0.16) | 0.99 (0.91, 1.06) |
|  | 2018 | 156.73 (156.39, 157.06) | 0.09 (0.08, 0.10) | 0.57 (0.52, 0.63) |
|  | 2019 | 161.94 (161.60, 162.28) | 0.08 (0.08, 0.09) | 0.52 (0.46, 0.57) |

CI, Confidence interval. **
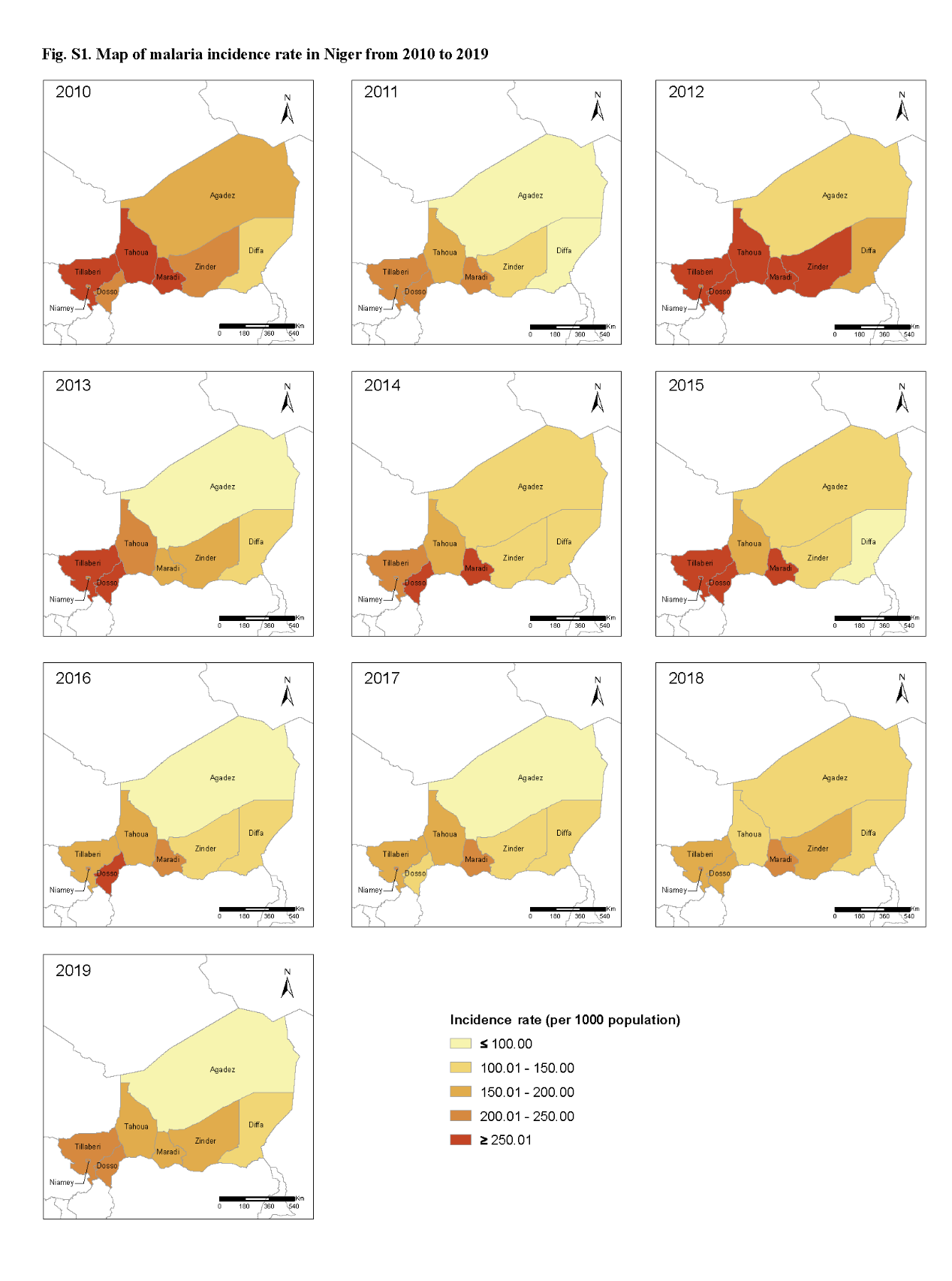
**

**Fig. S1. Map of malaria incidence rate in Niger from 2010 to 2019.**

**
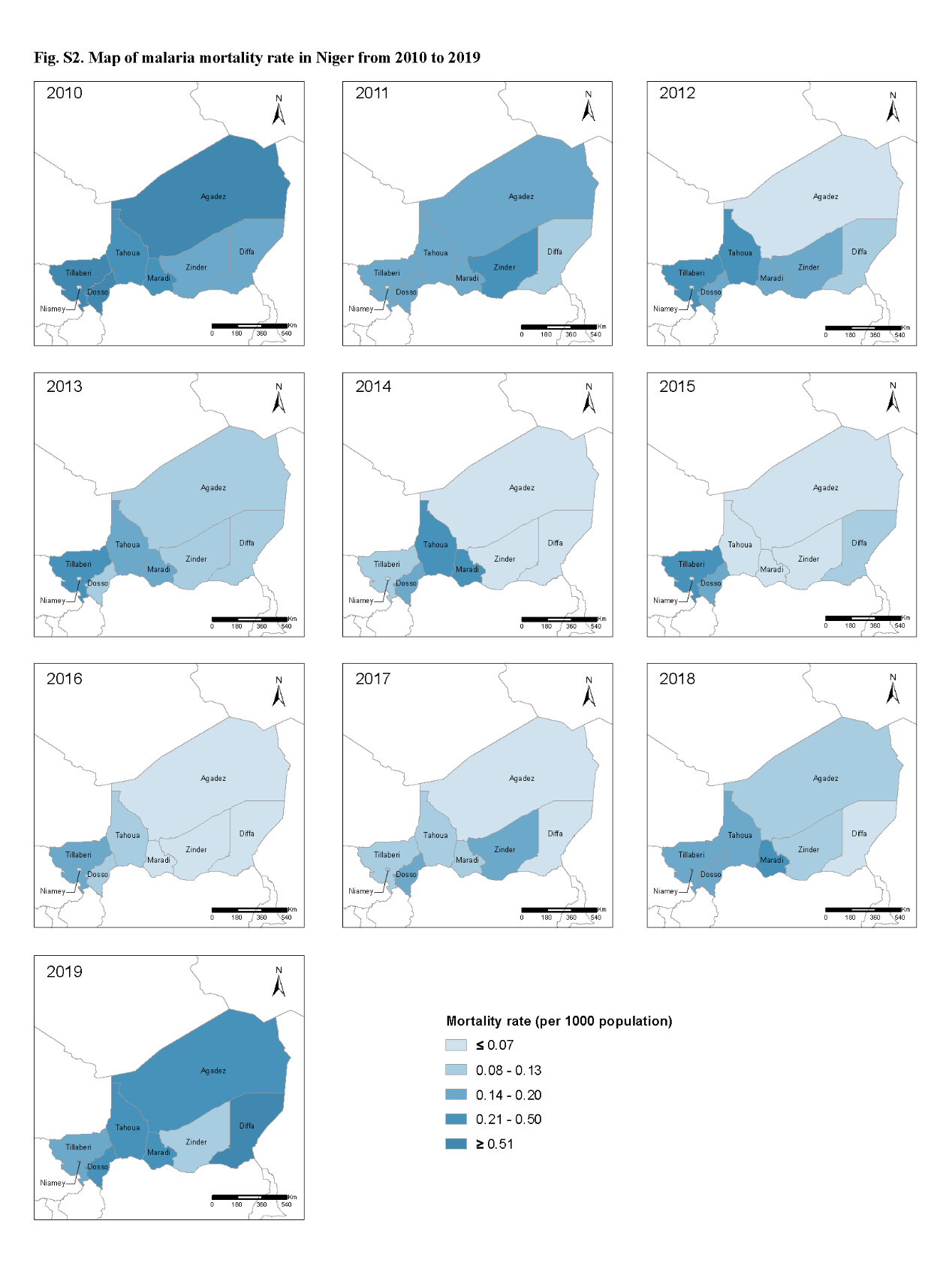
**

**Fig. S2. Map of malaria mortality rate in Niger from 2010 to 2019.
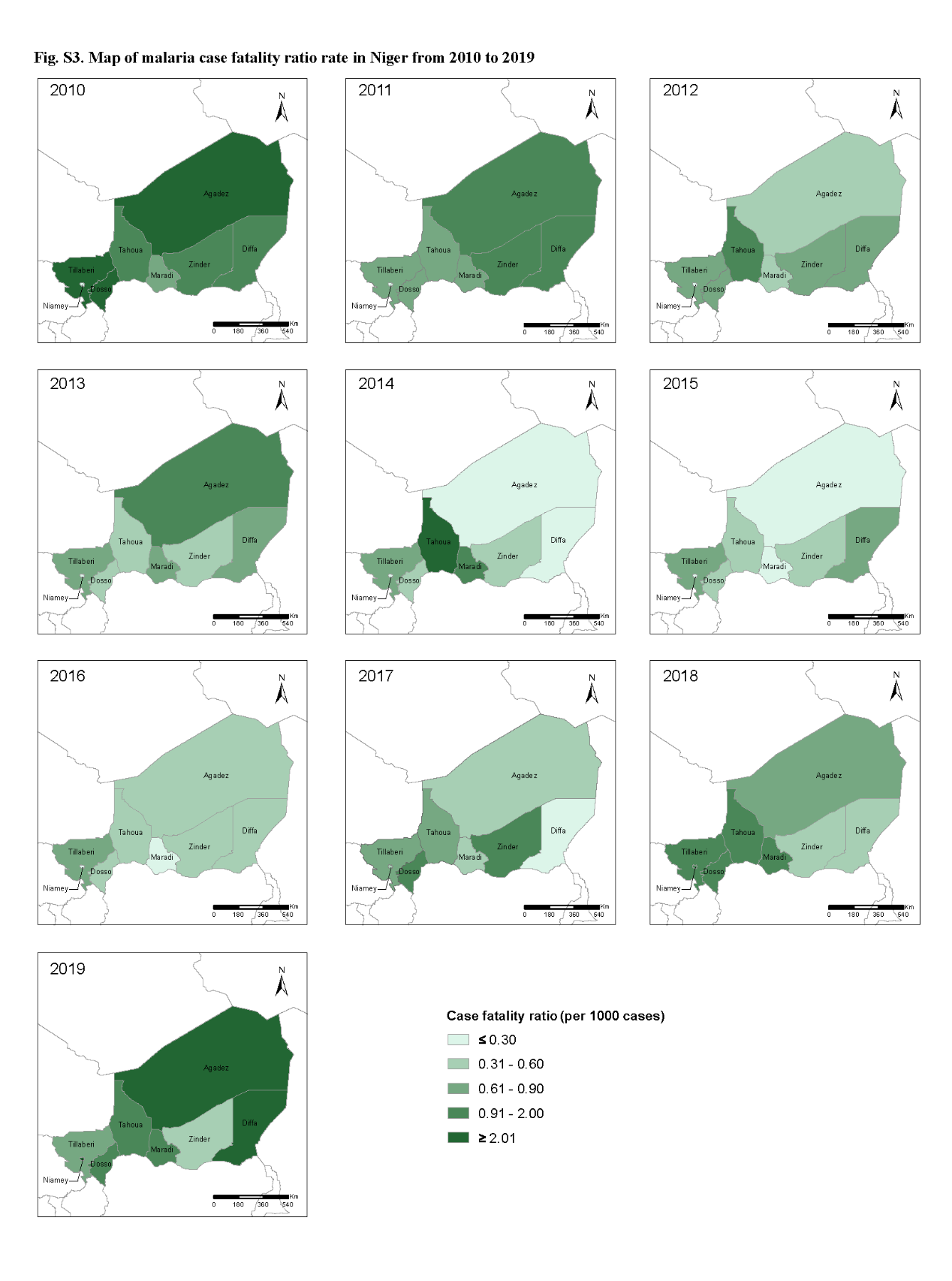
**

**Fig. S3. Map of malaria case fatality ratio rate in Niger from 2010 to 2019. Table S2. Annual epidemiological indicators of malaria by age group in Niger from 2010 to 2019.**

| **Year** | **Age group** | **No. of population at risk (%)** | **No. of malaria cases (%)** | **No. of malaria deaths (%)** | **Incidence rate, per 1,000 population** | **Mortality rate, per 1,000 population** | **Case fatality ratio, per 1,000 cases** |
| --- | --- | --- | --- | --- | --- | --- | --- |
| Average |  |  |  |  |  |  |  |
|  | < 1 | 792,490.90 (4.27) | 559,816.70 (15.11) | 417.40 (12.61) | 706.40 | 0.53 | 0.75 |
|  | 1–4 | 3,127,606.10 (16.83) | 1,640,510.60 (44.27) | 1960.40 (59.21) | 524.53 | 0.63 | 1.19 |
|  | 5–9 | 3,083,732.00 (16.60) | 417,496.30 (11.27) | 427.70 (12.92) | 135.39 | 0.14 | 1.02 |
|  | 10–24 | 5,915,470.40 (31.84) | 539,259.90 (14.55) | 267.40 (8.08) | 91.16 | 0.05 | 0.50 |
|  | ≥ 25 | 5,659,582.30 (30.46) | 548,214.70 (14.80) | 238.20 (7.19) | 96.86 | 0.04 | 0.43 |
| 2010 |  |  |  |  |  |  |  |
|  | < 1 | 615,430 (3.91) | 710,231 (18.10) | 1085 (16.29) | 1154.04 | 1.76 | 1.53 |
|  | 1–4 | 2,829,031 (17.98) | 1,930,627 (49.19) | 3784 (56.80) | 682.43 | 1.34 | 1.96 |
|  | 5–9 | 2,491,323 (15.83) | 407,199 (10.38) | 778 (11.68) | 163.45 | 0.31 | 1.91 |
|  | 10–24 | 4,909,236 (31.20) | 448,872 (11.44) | 448 (6.72) | 91.43 | 0.09 | 1.00 |
|  | ≥ 25 | 4,888,969 (31.07) | 427,601 (10.90) | 567 (8.51) | 87.46 | 0.12 | 1.33 |
| 2011 |  |  |  |  |  |  |  |
|  | < 1 | 636,654 (3.91) | 540,900 (17.46) | 287 (10.54) | 849.6 | 0.45 | 0.53 |
|  | 1–4 | 2,896,581 (17.80) | 1,481,537 (47.84) | 1772 (65.10) | 511.48 | 0.61 | 1.20 |
|  | 5–9 | 2,547,849 (15.66) | 323,537 (10.45) | 271 (9.96) | 126.98 | 0.11 | 0.84 |
|  | 10–24 | 5,158,790 (31.70) | 324,934 (10.49) | 139 (5.11) | 62.99 | 0.03 | 0.43 |
|  | ≥ 25 | 5,031,811 (30.92) | 426,269 (13.76) | 253 (9.29) | 84.71 | 0.05 | 0.59 |
| 2012 |  |  |  |  |  |  |  |
|  | < 1 | 657,867 (3.91) | 724,042 (16.11) | 325 (10.48) | 1100.59 | 0.49 | 0.45 |
|  | 1–4 | 2,972,312 (17.66) | 2,106,643 (46.88) | 2146 (69.23) | 708.76 | 0.72 | 1.02 |
|  | 5–9 | 2,613,493 (15.53) | 475,434 (10.58) | 382 (12.32) | 181.92 | 0.15 | 0.80 |
|  | 10–24 | 5,403,973 (32.11) | 479,736 (10.68) | 110 (3.55) | 88.77 | 0.02 | 0.23 |
|  | ≥ 25 | 5,181,404 (30.79) | 707,549 (15.75) | 137 (4.42) | 136.56 | 0.03 | 0.19 |
| 2013 |  |  |  |  |  |  |  |
|  | < 1 | 680,038 (3.91) | 63,0973 (17.53) | 191 (8.65) | 927.85 | 0.28 | 0.30 |
|  | 1–4 | 3,052,347 (17.54) | 1,482,574 (41.18) | 1494 (67.63) | 485.72 | 0.49 | 1.01 |
|  | 5–9 | 2,680,809 (15.40) | 440,262 (12.23) | 249 (11.27) | 164.23 | 0.09 | 0.57 |
|  | 10–24 | 5,651,863 (32.47) | 460,474 (12.79) | 140 (6.34) | 81.47 | 0.02 | 0.30 |
|  | ≥ 25 | 5,339,031 (30.68) | 585,905 (16.27) | 135 (6.11) | 109.74 | 0.03 | 0.23 |
| 2014 |  |  |  |  |  |  |  |
|  | < 1 | 823,617 (4.65) | 554,497 (15.47) | 341 (10.09) | 673.25 | 0.41 | 0.61 |
|  | 1–4 | 2,996,957 (16.94) | 1,655,431 (46.18) | 2440 (72.19) | 552.37 | 0.81 | 1.47 |
|  | 5–9 | 3,093,662 (17.48) | 383,670 (10.70) | 269 (7.96) | 124.02 | 0.09 | 0.70 |
|  | 10–24 | 5,377,827 (30.39) | 450,621 (12.57) | 207 (6.12) | 83.79 | 0.04 | 0.46 |
|  | ≥ 25 | 5,403,762 (30.54) | 540,188 (15.07) | 123 (3.64) | 99.97 | 0.02 | 0.23 |
| 2015 |  |  |  |  |  |  |  |
|  | < 1 | 856,099 (4.63) | 524,654 (14.11) | 191 (11.58) | 612.84 | 0.22 | 0.36 |
|  | 1–4 | 3,115,954 (16.87) | 1,653,908 (44.48) | 1119 (67.82) | 530.79 | 0.36 | 0.68 |
|  | 5–9 | 3,216,220 (17.41) | 456,242 (12.27) | 213 (12.91) | 141.86 | 0.07 | 0.47 |
|  | 10–24 | 5,664,156 (30.66) | 489,548 (13.16) | 78 (4.73) | 86.43 | 0.01 | 0.16 |
|  | ≥ 25 | 5,620,990 (30.43) | 594,333 (15.98) | 49 (2.97) | 105.73 | 0.01 | 0.08 |
| 2016 |  |  |  |  |  |  |  |
|  | < 1 | 889,909 (4.65) | 501,694 (14.14) | 174 (11.15) | 563.76 | 0.20 | 0.35 |
|  | 1–4 | 3,239,850 (16.95) | 1,551,419 (43.74) | 1031 (66.05) | 478.86 | 0.32 | 0.66 |
|  | 5–9 | 3,343,812 (17.49) | 446,137 (12.58) | 221 (14.16) | 133.42 | 0.07 | 0.50 |
|  | 10–24 | 5,808,726 (30.38) | 471,374 (13.29) | 77 (4.93) | 81.15 | 0.01 | 0.16 |
|  | ≥ 25 | 5,835,119 (30.52) | 576,461 (16.25) | 58 (3.72) | 98.79 | 0.01 | 0.10 |
| 2017 |  |  |  |  |  |  |  |
|  | < 1 | 894,079 (4.31) | 452,105 (13.67) | 387 (14.48) | 505.67 | 0.43 | 0.86 |
|  | 1–4 | 3,312,632 (15.97) | 1385,056 (41.88) | 1370 (51.27) | 418.11 | 0.41 | 0.99 |
|  | 5–9 | 3,600,468 (17.36) | 348,251 (10.53) | 441 (16.50) | 96.72 | 0.12 | 1.27 |
|  | 10–24 | 6,786,623 (32.71) | 646,584 (19.55) | 284 (10.63) | 95.27 | 0.04 | 0.44 |
|  | ≥ 25 | 6,152,013 (29.65) | 475,215 (14.37) | 190 (7.11) | 77.25 | 0.03 | 0.40 |
| 2018 |  |  |  |  |  |  |  |
|  | < 1 | 930,978 (4.32) | 472,142 (12.84) | 583 (15.45) | 507.15 | 0.63 | 1.23 |
|  | 1–4 | 3,431,074 (15.90) | 1,536,159 (41.78) | 1974 (52.31) | 447.72 | 0.58 | 1.29 |
|  | 5–9 | 3,636,353 (16.85) | 407,649 (11.09) | 602 (15.95) | 112.10 | 0.17 | 1.48 |
|  | 10–24 | 7,189,767 (33.32) | 752,544 (20.47) | 425 (11.26) | 104.67 | 0.06 | 0.56 |
|  | ≥ 25 | 6,386,567 (29.60) | 508,451 (13.83) | 190 (5.03) | 79.61 | 0.03 | 0.37 |
| 2019 |  |  |  |  |  |  |  |
|  | < 1 | 940,238 (4.28) | 486,929 (11.87) | 610 (11.34) | 517.88 | 0.65 | 1.25 |
|  | 1–4 | 3,429,323 (15.63) | 1,621,752 (39.52) | 2474 (45.98) | 472.91 | 0.72 | 1.53 |
|  | 5–9 | 3,613,331 (16.47) | 486,582 (11.86) | 851 (15.81) | 134.66 | 0.24 | 1.75 |
|  | 10–24 | 7,203,743 (32.83) | 867,912 (21.15) | 766 (14.24) | 120.48 | 0.11 | 0.88 |
|  | ≥ 25 | 6,756,157 (30.79) | 640,175 (15.60) | 680 (12.64) | 94.75 | 0.10 | 1.06 |


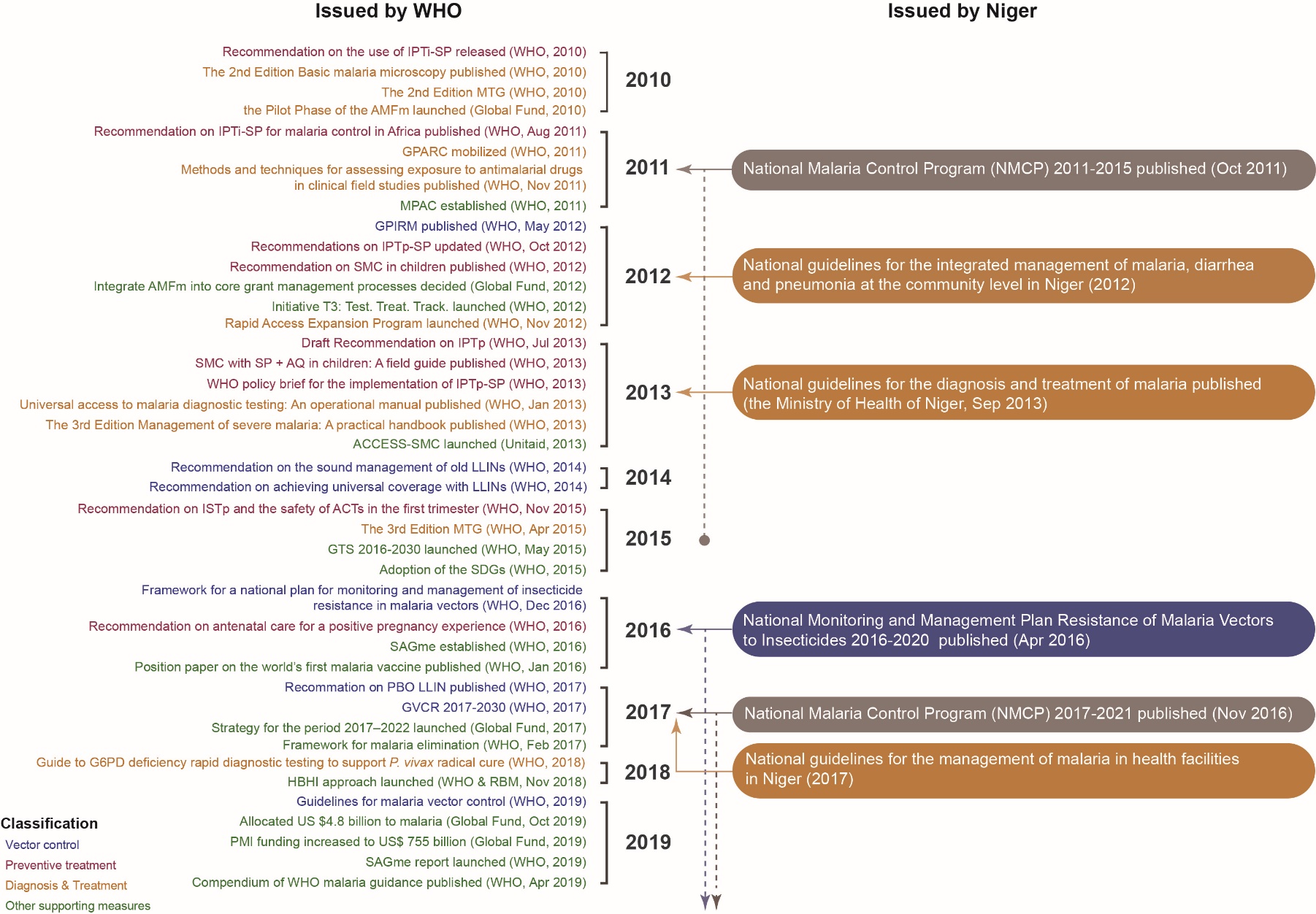


**Fig. S4. Interventions issued by the World Health Organization (WHO) and Niger.**

Interventions for malaria control and elimination were divided into four categories, including vector control, preventive treatment, early diagnosis and prompt treatment, and other supporting measures, showing in different colors.

IPTi, intermittent preventive treatment in infants; SP, sulfadoxine-pyrimethamine; MTG, Guidelines for the treatment of malaria; AMFm, Affordable Medicines Facility-malaria; GPARC, Global Plan for Artemisinin Resistance Containment; MPAC, Malaria Policy Advisory Committee; GPIRM, Global Plan for Insecticide Resistance Management in Malaria; IPTp, intermittent preventive treatment of malaria in pregnancy; SMC, seasonal malaria chemoprevention; AQ, amodiaquine; LLIN, long-lasting insecticidal net; ISTp, intermittent screening and treatment in pregnancy; ACT, artemisinin-based combination therapy; GTS, Global Technical Strategy for Malaria; SDG, Sustainable Development Goal; SAGme, Strategic Advisory Group on Malaria Eradication; PBO, piperonyl butoxide; GVCR, Global Vector Control Response; G6PD, glucose-6-phosphate dehydrogenase; *P. vivax,* *Plasmodium vivax*; HBH, High Burden to High Impact; US, United States; PMI, President’s Malaria Initiative.
